# L-methylfolate, a new option in psychiatric treatment, would it be linked to psoriasis relapse?

**DOI:** 10.31744/einstein_journal/2020RC5522

**Published:** 2020-11-18

**Authors:** Rubens Pitliuk, Tatyanny Paula Pinto da Costa Santos Fucci

**Affiliations:** 1 Hospital Israelita Albert Einstein São PauloSP Brazil Hospital Israelita Albert Einstein, São Paulo, SP, Brazil.; 2 Nutricionista São PauloSP Brazil Nutricionista, São Paulo, SP, Brazil.

**Keywords:** Psoriasis, Methylenetetrahydrofolatereductase (NADPH2), Polymorphism, genetic, Depression, L-5-methyltetrahydrofolate, L-Methylfolate

## Abstract

We report a case of a 61-years-old woman in remission of psoriasis for 20 years. She presented recurrence of psoriasis in the form of plaques few days after taking L-methylfolate 15mg/day. The L-methylfolate was prescribed as an adjuvant for the treatment of depression in a patient with the methylenetetrahydrofolate reductase gene polymorphism (MTHFR).

## INTRODUCTION

Folate, a B vitamin, is essential to the brain for noradrenalin syntheses, serotonin, and dopamine. Three forms of folate are commonly used: folic acid, 5- methyltetrahydrofolate (5-MTHF, methylfolate and L-methylfolate) and folic acid.^(^^1^^)^

The biologically activated form of folate, the L-methylfolate, is the only folate metabolism that cross the blood-brain barrier, and regulate the formation of tetrahydrobiopterin cofactor (BH4).^(^^2^^)^ The BH4 is a pterin that acts as a mandatory cofactor for the activity of hydroxylase phenylalanine, tyrosine hydroxylase and tryptophan hydroxylase, which catalysis the initial stages of degradation of phenylalanine in the liver, therefore, as well as the limitation stages of biosynthesis of dopamine neurotransmitters and serotonin.^(^^3^^)^

Because folate performs a crucial role in the central nervous system, it is believed that individuals with reduced levels can achieve higher rates of depression and poor response to antidepressants treatment.^(^^2^^)^

Individuals with polymorphism of MTHFR gene of C677T and A1298C types, both associated with metabolism or folate absorption, may better benefit from 5-MTHF, instead to the folic acid.^(^^4^^)^

Some findings add to evidences that implicate in methylation disorders of nervous system in biology of some forms of mental disease. In a sample of 123 psychiatric patients (diagnostic of higher depression or schizophrenia), 41 had borderline deficiency or defining folate (serum folate below 200µg/L). In this double-blind placebo study a total of 15mg daily of L-methylfolate was administered for 6 months, in addition to the psychotropic coadjuvant treatment. Patients presented significant clinical and social recovery to the use of L-methylfolate *versus* placebo.^(^^5^^)^

Other studies added 5-MTHF to antidepressant to people with low folate concentration or to individuals whose depression did not respond to drugs. The increase of folate during treatment with antidepressant may improve the efficacy of antidepressant not responding and allows that those that responded partly to the monotherapy with antidepressant to reach abetter response.^(^^6^^)^

Psoriasis, common inflammatory systemic disorders, affects mainly the skin and nails and, occasionally, joints. The prevalence of psoriasis in different population ranges between 0 and 12%.^(^^7^^)^

Methotrexate drug has been indicated for erythrodermic psoriasis, moderate to severe arthropathic psoriasis, acute pustular psoriasis, severe plaque psoriasis or disabling psoriasis and to cases with poor response to phototherapy and/or retinoids.^(^^8^^)^

There are suggestions in the published literature that L-methylfolate may cause reduction of efficacy of methotrexate for treatment of psoriasis, such as the case of a 53-yeard-old man who presented worsening of psoriasis after it had solved almost completely 2 years before, although under continuing therapy with methotrexate. In addition, he had recurrence of psoriatic lesions and new psoriatic lesions covering 80% of his body, approximately 4 to 5 weeks after the beginning of L-methylfolate 15mg daily for depression treatment.^(^^9^^)^

Study submitted and approved by Research Ethics Committee of the *Hospital Israelita Albert Einstein* , and registered under the protocol number 3.930.986 and CAAE: 30171919.2.0000.0071.

## CASE REPORT

A 61-year-old white women, married, mother of two children, reported worsening of psoriasis symptoms after using L-methylfolate 15mg daily for coadjutant treatment of depression. She reported depression and psoriasis since she was 20 years old. The patient was without recurrent lesions of psoriasis since 41 years of age.

Due to the history of depression, she was prescribed to use lithium carbonate, however, because of psoriasis the substance was avoided. In 2000, she had improvement in the depression by taking venlafaxine 225 mg once a day. In 2016, because of humor oscillations, lamotrigine 100 mg to day was added and, to improve her sleeping quetiapine 50mg daily was prescribed. These were the current dosages, with improvement of the depression, quality of life and significant adverse effects.

In January 2019, polymorphism in MTHFR gene was detected, with mutation of heterozygous for C677T, and the patient received a prescription for 15mg daily from L-methylfolate. Before finishing the first week of the use of L-methylfolate, psoriatic lesions had appeared, which did not occurred for 20 years. The occurrence was reported in psychiatric that removed the medication, and, 4 days later, psoriatic lesions begun to recovery, and disappeared completely after few weeks. The patient remained without new changes up to beginning of 2020.

## DISCUSSION

After 20 years of being asymptomatic, there was recurrence of psoriasis with less than 1 week of the use of L-methylfolate 15mg daily. The patient reported not experience stress or recurrence of depression, which confirmed the stable picture. There were no changes in the patient's eating habit and medicines, expect to the L-methylfolate.

The importance of this report relays on the coadjuvant psychiatric therapy of high doses of L-methylfolate in patients with history of psoriasis. The present case seems to be the second case reported in the published literature that associate L-methylfolate and worsening of psoriasis.^(^^9^^)^ Differently from cases already reported, our patient did not use methotrexate concomitantly, therefore, disregarding the hypothesis of possible interaction between methotrexate and L-methylfolate.

The similarity between cases and possible correlation between L-methylfolate and psoriasis had drawn our attention, a fact that led to the hypothesis of high doses of L-methylfolate in direct relationship.

Although L-methylfolate 15mg has been formulated with 1mg of methylcobalamin, we believe that the first has a direct relationship and triggering role in psoriasis recurrence. Some reasons that may support this believe: close relationship with clinical cases already reported;^(^^9^^)^ high dosage of L-methylfolate, approximately 15 times to the tolerable upper intake levels (UL) for folate, described by the Food and Nutrition Board do Institute of Medicine;^(^^10^^)^ and the fact that methylcobalamin 1mg corresponds to commonly prescribed dosage for supplementation for B12 vitamin deficiency, which in this case was already used by patient without reporting of adverse effect.

## CONCLUSION

Considering the increase of cases treated with higher doses of L-methylfolate as coadjuvant for individuals with MTHFR gene polymorphism, the use of this substance must be carefully evaluated for patients with history of psoriasis.
