# Supplementary material for: L-methylfolate, a new option in psychiatric treatment, would it be linked to psoriasis relapse?
Source: Einstein (Sao Paulo). 2020 Nov 18;18:eRC5522. doi: 10.31744/einstein_journal/2020RC5522 (PMC7687916; doi:10.31744/einstein_journal/2020RC5522)
Supplement: Translation

# Translation: L-metilfolato, uma nova opção no tratamento psiquiátrico, estaria ligado à recaída de psoríase?

**Authors:** Rubens Pitliuk, Tatyanny Paula Pinto da Costa Santos Fucci

**Affiliations:** 1 São PauloSP Brasil Hospital Israelita Albert Einstein, São Paulo, SP, Brasil.; 2 São PauloSP Brasil Nutricionista, São Paulo, SP, Brasil.

**Keywords:** Psoríase, Metilenotetrahidrofolato redutase (NADPH2), Polimorfismo genético, Depressão, L-5-methyltetrahydrofolate, L-Metilfolato

## Abstract

Paciente do sexo feminino, 61 anos, em remissão da psoríase por 20 anos. Apresentou recidiva de psoríase em forma de placas poucos dias após início de tratamento L-metilfolato na dose diária de 15mg. O L-metilfolato foi prescrito como terapêutica coadjuvante para tratamento de depressão em paciente portadora do polimorfismo do gene metilenotetrahidrofolato redutase.

## INTRODUÇÃO

O folato, uma vitamina B, é necessário ao cérebro para síntese de noradrenalina, serotonina e dopamina. Três formas de folato são comumente usadas: ácido fólico, 5-metiltetrahidrofolato (5-MTHF, metilfolato e L-metilfolato) e ácido folínico.^(^^1^^)^

A forma biologicamente ativa de folato, o L-metilfolato, é o único metabólito do folato que atravessa a barreira hematoencefálica, regulando a formação do cofator tetraidrobiopterina (BH4).^(^^2^^)^A BH4 é uma pterína que atua como cofator obrigatório para a atividade das enzimas fenilalanina hidroxilase, tirosina hidroxilase e triptofano hidroxilase, que catalisam as etapas iniciais da degradação da fenilalanina no fígado, assim como as etapas limitantes da biossíntese dos neurotransmissores dopamina e serotonina.^(^^3^^)^

Como o folato desempenha papel crucial no sistema nervoso central, propõe-se que os indivíduos com níveis reduzidos possam ter taxas mais altas de depressão e resposta fraca ao tratamento com antidepressivos.^(^^2^^)^

Portadores de polimorfismo do gene MTHFR dos tipos C677T e A1298C, relacionados ao metabolismo ou à absorção do folato, podem se beneficiar melhor do 5-MTHF, ao invés do ácido fólico.^(^^4^^)^

Algumas descobertas se somam às evidências que implicam em distúrbios da metilação do sistema nervoso na biologia de algumas formas de doença mental. Em amostra de 123 pacientes psiquiátricos (diagnóstico de depressão maior ou esquizofrenia), 41 tinham deficiência limiar ou definida de folato (folato sérico abaixo de 200µg/L). Nesse estudo duplo-cego, placebo, administraram-se 15mg ao dia de L-metilfolato, durante 6 meses, além de tratamento psicotrópico coadjuvante. Os pacientes apresentaram significativa recuperação clínica e social ao uso de L-metilfolato *versus* placebo.^(^^5^^)^

Outros estudos adicionaram 5-MTHF a antidepressivos para pessoas com baixo teor de folato ou em indivíduos cuja depressão não era responsiva às drogas. O aumento de folato durante o tratamento com antidepressivos poderia melhorar a eficácia dos antidepressivos em não respondedores e permitir que aqueles que respondem parcialmente à monoterapia com antidepressivos alcancem melhor resposta.^(^^6^^)^

A psoríase, distúrbio inflamatório sistêmico comum, afeta principalmente a pele, as unhas e, ocasionalmente, as articulações. A prevalência de psoríase em diferentes populações varia entre zero e 12%.^(^^7^^)^

O medicamento metotrexato vem sendo indicado para psoríase eritrodérmica, psoríase artropática moderada a grave, psoríase pustulosa aguda, psoríase em placas grave ou incapacitante e em casos com má resposta à fototerapia e/ou retinoides.^(^^8^^)^

Há sugestões na literatura de que o L-metilfolato pode causar diminuição da eficácia do metotrexato para tratamento da psoríase, como o caso de um homem de 53 anos que apresentou piora da psoríase após tê-la resolvido quase 100% nos 2 anos anteriores, apesar da continuação da terapia com metotrexato. Houve recorrência de lesões psoriáticas e novas lesões psoriáticas, cobrindo 80% de sua superfície corporal, aproximadamente 4 a 5 semanas depois que começou a usar L-metilfolato 15mg ao dia para tratamento da depressão.^(^^9^^)^

Estudo submetido e aprovado pelo Comitê de Ética em Pesquisa do Hospital Israelita Albert Einstein, e registrado com o número de protocolo 3.930.986 e CAAE: 30171919.2.0000.0071.

## RELATO DE CASO

Paciente do sexo feminino, 61 anos, caucasiana, casada, dois filhos, relatou piora dos sintomas da psoríase após início de L-metilfolato 15mg ao dia para tratamento coadjuvante de depressão. Relatava depressão e psoríase desde os 20 anos. Desde os 41 anos, a paciente estava sem recidivas das lesões psoriáticas.

Pelo histórico de suas depressões, teve indicação de carbonato de lítio, mas foi evitado devido à psoríase. Em 2000, apresentou melhora do quadro depressivo, com venlafaxina 225mg ao dia. Em 2016, devido a oscilações de humor, foi acrescida lamotrigina 100mg ao dia e, para melhora do sono, foi usada a quetiapina 50mg ao dia.

Essas eram as doses atuais, com melhora da depressão, da qualidade de vida e sem efeitos adversos significativos.

Em janeiro de 2019, detectou-se polimorfismo no gene MTHFR, com mutação heterozigótica no C677T, e a paciente recebeu prescrição de 15mg ao dia de L-metilfolato. Antes do término da primeira semana de uso do L-metilfolato, surgiram lesões psoriáticas, que há 20 anos não se manifestavam. O transcorrido foi relatado ao psiquiatra, que retirou a medicação, e, 4 dias após, as lesões psoriáticas começaram a remitir, desaparecendo completamente após algumas semanas. A paciente permanecia sem novos registros de manifestações até início de 2020.

## DISCUSSÃO

Depois de 20 anos assintomática, houve recidiva da psoríase com menos de 1 semana de uso do L-metilfolato 15mg ao dia. A paciente relatou não passar por nenhum estresse e nem recaída da depressão, confirmando quadro estável. Não houve mudança no hábito alimentar e medicamentoso, além do L-metilfolato.

A importância deste relato refere-se à terapêutica psiquiátrica coadjuvante de altas doses de L-metilfolato em pacientes com histórico de psoríase. O presente caso deve ser o segundo relatado que associa L-metilfolato e piora de psoríase.^(^^9^^)^Diferentemente do caso já relatado, a paciente deste caso não fez uso concomitante de metotrexato, descartando a hipótese de possível interação entre este e L-metilfolato.

A semelhança entre os casos e a possível correlação entre L-metilfolato e psoríase despertaram nossa atenção, o que levanta a hipótese da alta dose de L-metilfolato em relação direta.

Embora 15mg de L-metilfolato tenham sido formuladas com 1mg de metilcobalamina, acreditamos que o primeiro tenha relação direta e papel desencadeante na recidiva da psoríase. Alguns motivos nos convergem a esse pensamento: estreita relação com o caso clínico já relatado;^(^^9^^)^dosagem alta de L-metilfolato, aproximadamente 15 vezes a *tolerable upper intake levels* (UL) de folate, descrita pela *Food and Nutrition Board* do *Institute of Medicine* ;^(^^10^^)^e o fato de a metilcobalamina 1mg corresponder à dose comumente prescrita na suplementação para insuficiência ou deficiência da vitamina B12, já utilizada pela paciente sem relato de efeito adverso.

## CONCLUSÃO

Com o aumento de casos tratados com doses elevadas de L-metilfolato como coadjuvante em portadores de polimorfismo do gene MTHFR, seu uso deve ser cuidadosamente avaliado em pacientes com histórico de psoríase.
